# Chemical Cues Released by an Alien Invasive Aquatic Gastropod Drive Its Invasion Success

**DOI:** 10.1371/journal.pone.0064071

**Published:** 2013-05-15

**Authors:** Jacqueline L. Raw, Nelson A. F. Miranda, Renzo Perissinotto

**Affiliations:** School of Life Sciences, University of KwaZulu-Natal, Durban, KwaZulu-Natal, South Africa; University of Western Ontario, Canada

## Abstract

**Background:**

Chemical cues provide aquatic organisms with sensory information that guides behavioural responses and thus interactions among themselves, each other and the environment. Chemical cues are considered important for predator avoidance, foraging, larval settlement and broadcast spawning in aquatic environments. However, the significance of their role as drivers of direct interactions between heterospecifics has been largely overlooked.

**Methodology/Principal Findings:**

A video camera and a demarcated arena were used *in situ* to record behavioural responses of three native gastropod species, *Assiminea* cf. *capensis*, *Melanoides tuberculata* and *Coriandria durbanensis*, exposed to treatments representing chemical cues released by a non-native invasive gastropod, *Tarebia granifera*. The responses were measured quantitatively as displacement and orientation of movement at locations in St Lucia Estuary, within the iSimangaliso Wetland Park, a UNESCO World Heritage Site on the east coast of South Africa. All native gastropods exhibited a negative taxis response to chemical cues released by *T. granifera*, while *T. granifera* individuals responded randomly to conspecifics. Displacement was measured relative to the source of the extract, the number of steps taken were determined with path analysis and orientation was determined from the mean (±95% CIs) turning angles, with significant negative turning angles representing negative taxis. Responses to treatments corresponding to the environment and conspecifics were random and undirected, indicating kinesis.

**Conclusion/Significance:**

This study presents evidence for interactions driven by chemical cues between a non-native invasive gastropod and several gastropods native to South Africa. The results indicate that chemical cues can facilitate invasion success as the behavioural response of native gastropods is to move away allowing additional food and space resources to become available to *T. granifera*.

## Introduction

Biological invasions, although considered a major component of anthropogenic global change, also provide an opportunity to study ecological principles which govern the coexistence of populations within communities [1]. Studies of biotic interactions such as predation, competition and facilitation between non-native and native species can translate to a better understanding of the relationships which exist among established native species [2]. Classic works considered exploitative competition as the primary biotic factor influencing the success of non-native species in new environments [3]. However, recent studies have emphasised interference competition and facilitation [4], [5], as well as the importance of environmental factors that together may contribute towards invasion resistance [6].

Biotic interactions remain an important component of invasion success [2] and are generally investigated at the level of the individual by quantifying behavioural responses [7]. Research has focussed on the interactions between agonistic competitors [8] and novel predator-prey relationships [9]. These studies have quantified behavioural responses in terms of activity levels, aggression, dominance and searching for refuge. However, this classification has not accounted for behavioural responses of individuals prior to confrontation, or for species that are not naturally aggressive. In order to assess the other facets of this phenomenon, it is necessary to examine communication as a function of behavioural responses [10]. Various types of communication exist among organisms and thus both the concept and definition have been scientifically debated since the 1960s [11], [12], [13]. Despite the conceptual debate, chemical communication among organisms was recognized early-on as an important component influencing behaviour [14], [15].

The importance of chemical cues in structuring aquatic communities has been addressed extensively by empirical studies [16]. Responses in aquatic organisms have been demonstrated for chemical cues corresponding to natural predators [17], food sources [18] and conspecific alarm cues [19]. These methods have been applied to investigate biotic interactions between native and non-native species with emphasis on naïve predator-prey interactions [20], [21], [22]. This study provides empirical support for chemical cues as a driver of important biotic interactions between native and non-native species.

Chemoreception has been comprehensively studied in gastropods [23] and many representatives of this class have become non-native invasive species of global importance [24]. Considering these two factors, gastropods have become the focus of behavioural response studies involving interactions mediated by chemical cues. As chemoreception is known to guide locomotion [23], empirical studies have quantified behavioural responses in terms of movement [25]. The aim of this study was to determine whether chemical cues released by a non-native gastropod, *Tarebia granifera*, drive interactions with three native gastropod species, *Assiminea* cf. *capensis*, *Melanoides tuberculata* and *Coriandria durbanensis*, by affecting their behaviour and thus facilitating its invasion success.

*Tarebia granifera*, a freshwater gastropod endemic to south-east Asia, has invaded many tropical and sub-tropical parts of the world [26] and has most recently become established at the expense of native species in areas of the iSimangaliso Wetland Park and many other shallow water ecosystems in KwaZulu-Natal, South Africa [27]. Generally it has been assumed that *T. granifera* displaces native gastropods through exploitative competition. However, as food resources are not limiting in iSimangaliso [28], it was hypothesized that chemical cues are involved in displacement interactions between *T. granifera* and native gastropod species.

## Materials and Methods

### Ethics Statement

All necessary permits were obtained from the iSimangaliso Wetland Park Authority for the described field studies at each location, under a Research Agreement for the project titled “Climate Change and the Management of KZN Estuaries: St Lucia Estuary”.

### Gastropods and Experiment Sites

**Figure 1:**
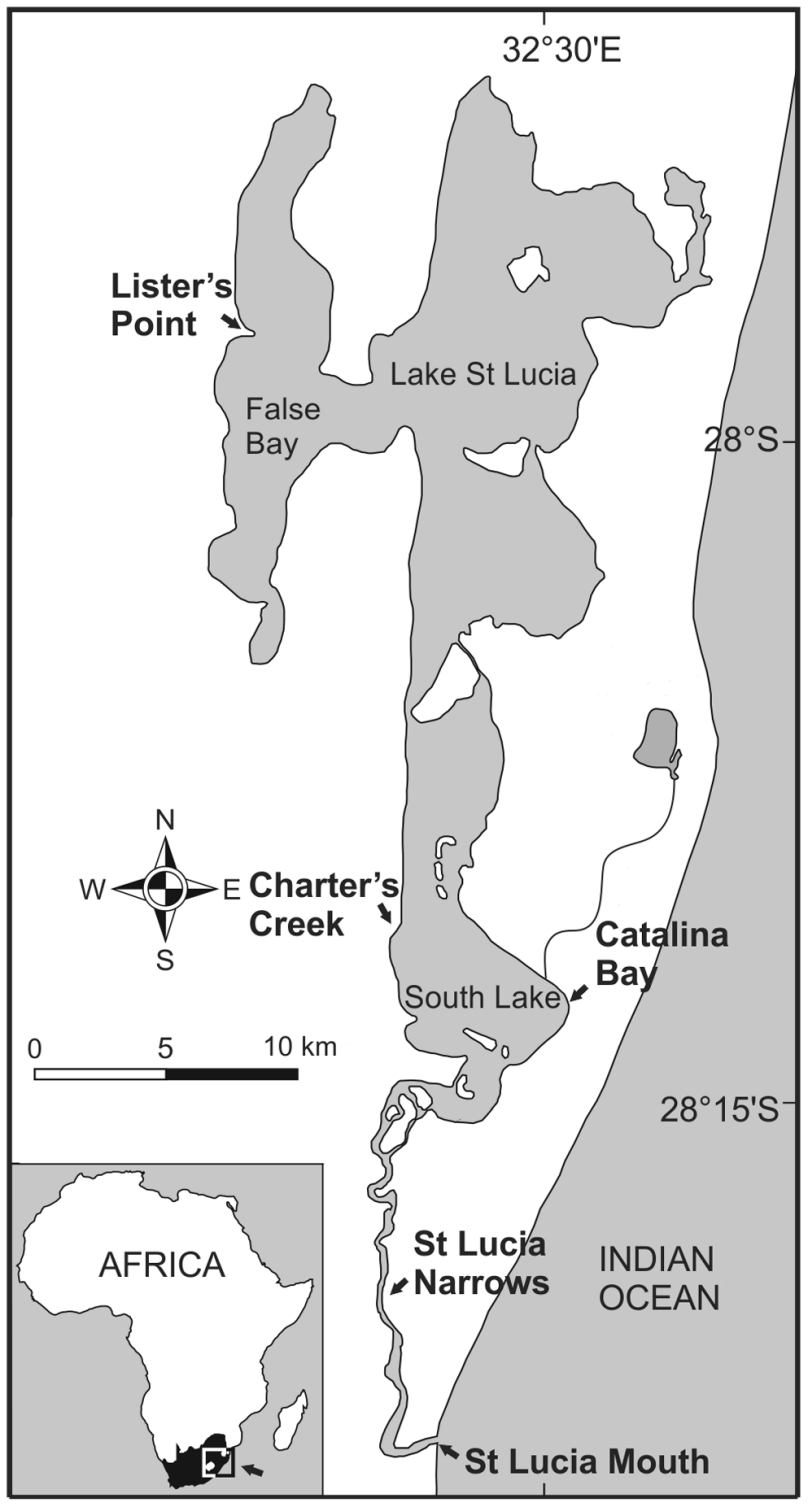
Locations of *in situ* experiments. Map of St Lucia Estuary, which is one of three Ramsar Wetlands of International Importance within the iSimangaliso Wetland Park in KwaZulu-Natal, South Africa (a UNESCO World Heritage Site since 1999).

The behavioural responses of the three native gastropod species, *Assiminea* cf. *capensis*, *Melanoides tuberculata* and *Coriandria durbanensis*, to chemical cues released by *T. granifera* were quantified independently in terms of movement and orientation following a protocol adapted from Wollerman et al. [25]. The experiments for this study were carried out in March 2012 at Charter’s Creek (28°12′3.29′′S 32°25′6.26′′E) and Catalina Bay (28°13′28.89′′S 32°29′11.79′′E) and in July 2012 at Lister’s Point (27°58′22.30′′S 32°22′28.24′′E) and St Lucia Estuary Mouth (28°22′48.80′′S 32°25′18.63′′E) (Fig. 1) [29]. Salinity and temperature were measured with a YSI 6600-V2 multiprobe at each site.

Lake St Lucia, the largest estuarine lake in Africa [29], is located within the iSimangaliso Wetland Park, which is a UNESCO World Heritage Site. St Lucia has experienced low water levels following a decade of prolonged mouth closure [30] and diversion of the Mfolozi River away from the St Lucia Estuary. Consequently, a reverse salinity gradient has formed with the northernmost regions experiencing hypersaline conditions until very recently [31]. The South Lake region is substantially less saline than the northern reaches, as a result of freshwater seepage from aquifers along its eastern shores and inputs from rivers [31].

The non-native species, *Tarebia granifera*, despite being considered a freshwater caenogastropod, has a remarkable tolerance for moderately saline conditions of up to 30 [32] and has thus become successfully established in areas of the St Lucia Narrows (average salinity 13.8), as well as Catalina Bay (average salinity 3.6). *T. granifera* has however not yet been found at Charter’s Creek (average salinity 4.0) on the western shore of the South Lake, while the salinity (54.4) at Lister’s Point in False Bay is well beyond its physiological tolerance [32].

In the St Lucia Estuary, *A.* cf. *capensis* and *C. durbanensis* (previously collectively referred to as *A. bifasciata* or *A.* cf. *ovata*) have been reported to inhabit moderately saline to hypersaline environments with sandy substrata, where they are an important component of the benthos [33]. Experiments with *A.* cf. *capensis* (3–5 mm shell height (SH)) were carried out at Catalina Bay where *T. granifera* (8–10 mm SH) currently dominates the benthic invertebrate assemblage, as well as at Charter’s Creek where the non-native species has either not been introduced or has not yet become established. Experiments with *C. durbanensis* (2–4 mm SH) were carried out at Lister’s Point in the northern region of the system, where salinities are too high for *T. granifera*. Experiments with *M. tuberculata* (8–10 mm SH), which is a widespread freshwater species in South Africa [34], were carried out at the St Lucia Estuary Mouth, where *T. granifera* has not become established, although it has been reported in the adjacent Narrows (Fig. 1). Experiments were conducted on the species present at each site at the time of the experiments. The only overlapping distributions were between *A.* cf. *capensis* and *T. granifera* at Catalina Bay.

### Experiments and Data Analysis

Individual gastropods were exposed to chemical cue extracts that formed treatments of (1) conspecific cues, (2) cues released by the non-native species and (3) a control treatment representing general environmental cues at the time of the experiment. For sites where *T. granifera* does not occur, extracts were prepared with individuals collected from Catalina Bay and the salinity of the extracts was raised to that of the site prior to the experiment. Extracts were prepared in 2 L buckets with 300 ml of filtered water (Millex GP 0.2 µm) and gastropods collected from the respective site one day prior to the experiment. The gastropods were added to the buckets and kept for a fixed period to allow conditioning of the water with naturally-released chemical cues. Cue concentration was controlled for by conditioning the collected volume with the same biomass (wet weight) of gastropods for each treatment for a fixed period of time. Final densities were similar to those observed for each gastropod species in their respective habitats. During the conditioning period, gastropods were provided with naturally-occurring benthic algae collected *in situ* on which they fed *ad libitum*. The control treatment was prepared as above, but contained no gastropods. After conditioning, the water was once again filtered to remove any particulate organic wastes and accumulated microbes. A volume of 50 ml was retained and frozen for subsequent nutrient analysis while 8 drops of food colouring (Robertsons BLUE) was added to the remaining extract to aid monitoring of its delivery during the experiment.

Experiments were carried out in a tank (90×32×38 cm) using small amounts of filtered water (±1 cm depth) from the site. Gastropods were collected immediately prior to the experiment. An inverted video camera was used to record behavioural responses and a Perspex arena, with two concentric demarcated circles (diameters 10 and 20 cm), was used for reference. Extracts were continuously delivered to the centre of the arena at a rate of 1.5 ml/min by a titration burette mounted 5 cm above the centre of the arena. After two minutes of extract delivery, single individuals were placed on the edge of the inner circle, facing the source. The response was recorded for three minutes.

The response to each treatment was quantified by calculating the displacement (D) relative to the source of the extract for each individual snail. The displacement of a snail was calculated by subtracting the distance from the extract source to the endpoint of the pathway of the snail (E) from the distance from the source to the origin of the pathway of the snail (O). Thus, D is positive if the snail moved towards the source and negative if the snail moved away from the source. Differences between D for each treatment were tested with ANOVA. Differences between the numbers of steps taken by snails in each treatment (see below) were also tested using ANOVA. There were 5 species/locations (*A*. cf. *capensis* at Catalina Bay, *A*. cf. *capensis* at Charter’s Creek, *C*. *durbanensis* at Lister’s Point, *M*. *tuberculata* at St Lucia Estuary Mouth and *T*. *granifera* at Catalina Bay), 3 treatments (conspecific cue, non-native species cue and control) and 10 replicates per treatment. Out of the 150 individuals used in experiments, 5 were excluded from the dataset as they displayed unusual behaviour and/or impaired activity.

Path analysis was done according to the method of Benhaumou and Bovet [35]. The response pathway of the snails was reduced to a sequence of changes of direction, each with its own side-dependent turning angle (α). The side-dependent turning angle is the angle between the direction of snail movement and the direction of the source of the extract. The turning angle is positive if the snail turns toward the stimulus. If the snail turns away from the stimulus, the turning angle is negative. Turning angles therefore fall within the range of −180° to 180°. If a snail orients using kinesis, it is equally likely to turn towards (positive α) or away (negative α) from the source after each step, regardless of the direction of its previous step. In this case, the mean of the distribution of turning angles (mean α) will not be significantly different from 0. However, if the snail orients using taxis, it will consistently turn towards the source (positive taxis) or away from the source (negative taxis) and the mean α will be significantly different from 0. First, the mean turning angle (mean α) of each individual snail was calculated. The mean turning angles for each treatment were then averaged to determine the grand mean turning angle (grand mean α). Finally, a one-sample t-test (two-tailed) was used to compare each grand mean α to zero. To measure turning angles, the pathway of each snail was reduced to a number of discrete linear steps by reconstructing its pathway using a constant step length R [35]. Trials were conducted to determine an appropriate step length for discretizing snail pathways, using control treatments of each snail species. The path of each snail was discretized three times, with step lengths of 0.4, 0.8, and 1 cm. A mean α for each pathway was calculated using each step length, with a Kruskal–Wallis non-parametric analysis of variance showing no significantly different results between the three step lengths. A step length of 0.8 cm was chosen for all analyses because it produced a discretized pathway that was easy to measure and resulted in many turning angles. The number of steps taken by snails was determined according to the standardized step length and interpreted as a proxy for physical activity.

The pathways were traced, scanned and processed with ImageJ software. The 5 individual outliers contributed 6% of the path analysis data which were excluded from the analyses. All assumptions for parametric tests were satisfied. Data were analyzed and the “displacement” and “steps” graphs were generated in IBM SPSS v.19 for Windows. Turning angle graphs were generated with Corel Draw X5 for Windows.

## Results

**Figure 2:**
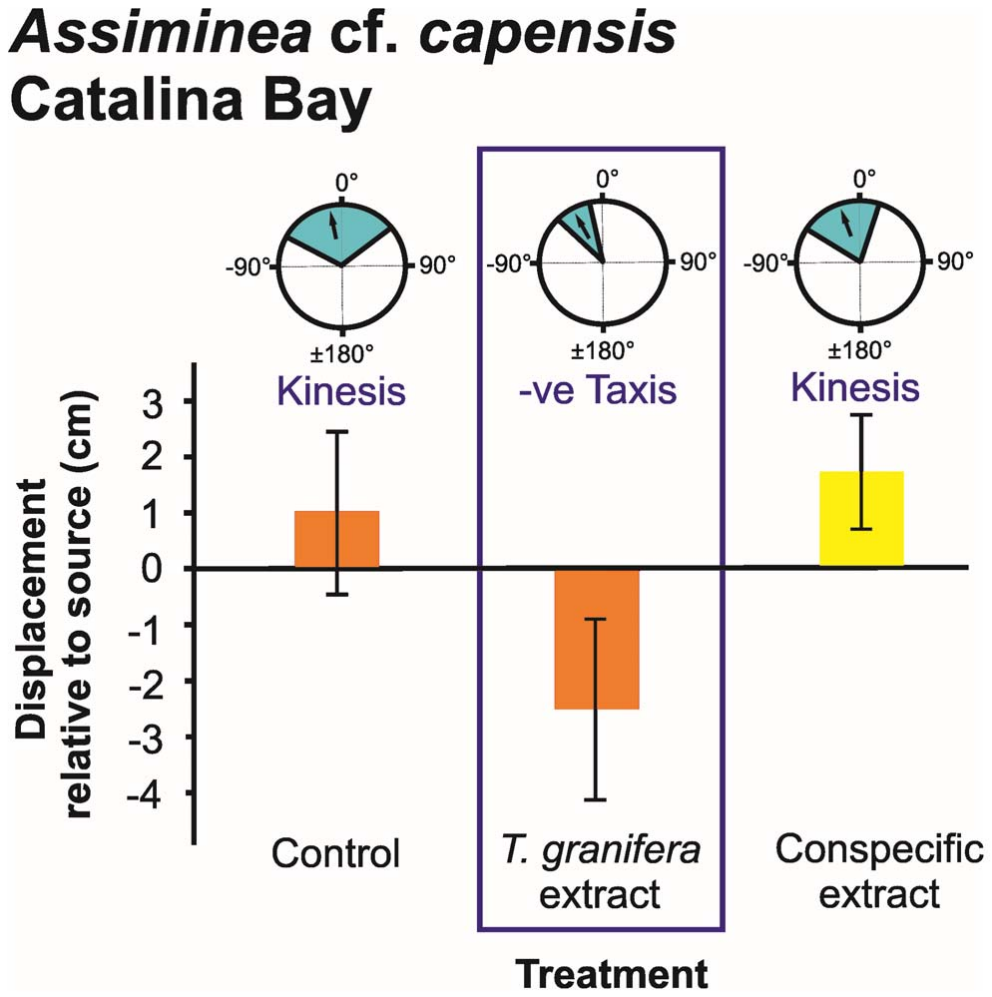
*Assiminea* cf. *capensis* orientation and movement responses to different chemical cue treatments at Catalina Bay. Three types of data are presented: (**1**) Mean turning angles distribution in response to chemical cue treatments (pie graphics). Arrows represent the overall mean for the treatment and shaded slices represent 95% CIs. Type of motion, i.e. taxis or kinesis, is indicated; (**2**) Mean displacement (± s.d.) of individuals relative to the source of chemical cue (bar graphics); (**3**) Average number of steps taken by individuals during the experiment (colour legend: yellow = 3 to 5 steps, orange = 6 to 9 steps, red = 10 to 13 steps). Treatments where a negative (−ve) taxis response occurred are demarcated. Individuals were tracked over a period of three minutes.

**Figure 3:**
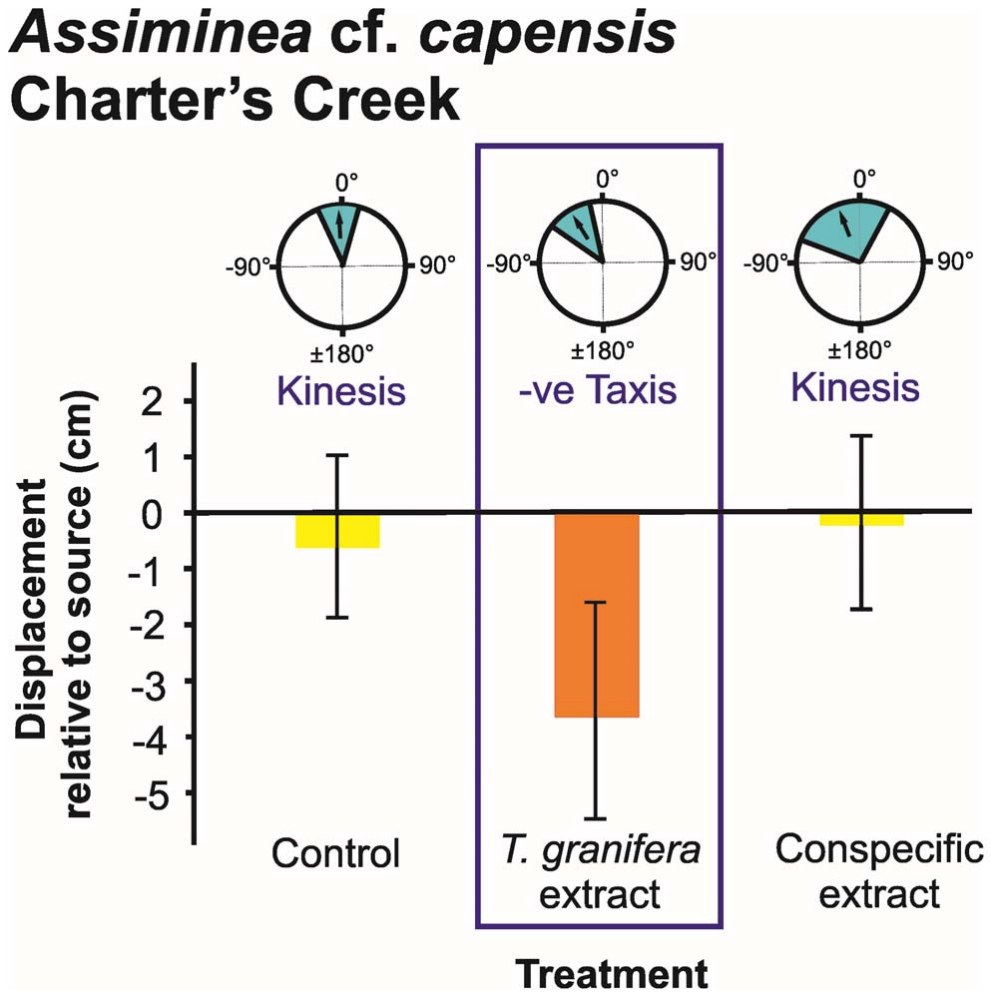
*Assiminea* cf. *capensis* orientation and movement responses to different chemical cue treatments at Charter’s Creek. Three types of data are presented: **refer to Fig. 2**. Treatments where a negative (−ve) taxis response occurred are demarcated. Individuals were tracked over a period of three minutes.

**Figure 4:**
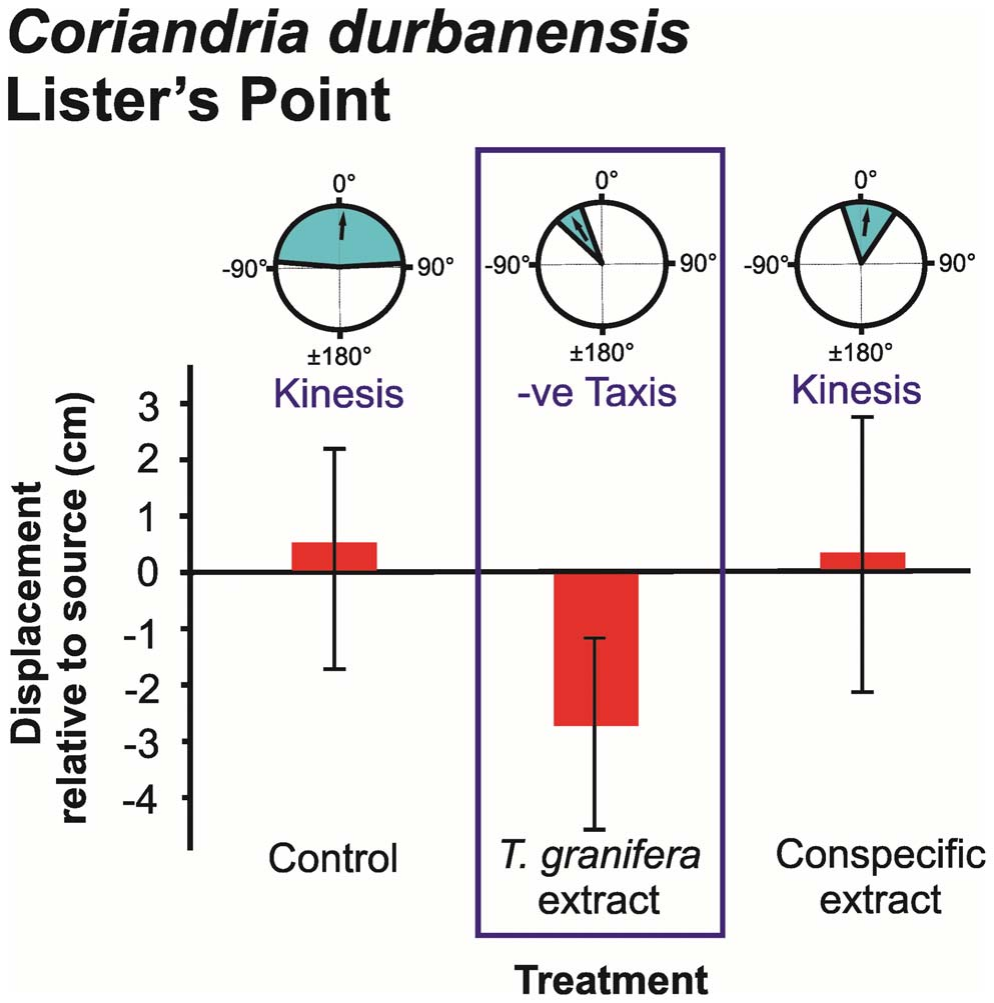
*Coriandria durbanensis* orientation and movement responses to different chemical cue treatments at Lister’s Point. Three types of data are presented: **refer to Fig. 2**. Treatments where a negative (−ve) taxis response occurred are demarcated. Individuals were tracked over a period of three minutes.

**Figure 5:**
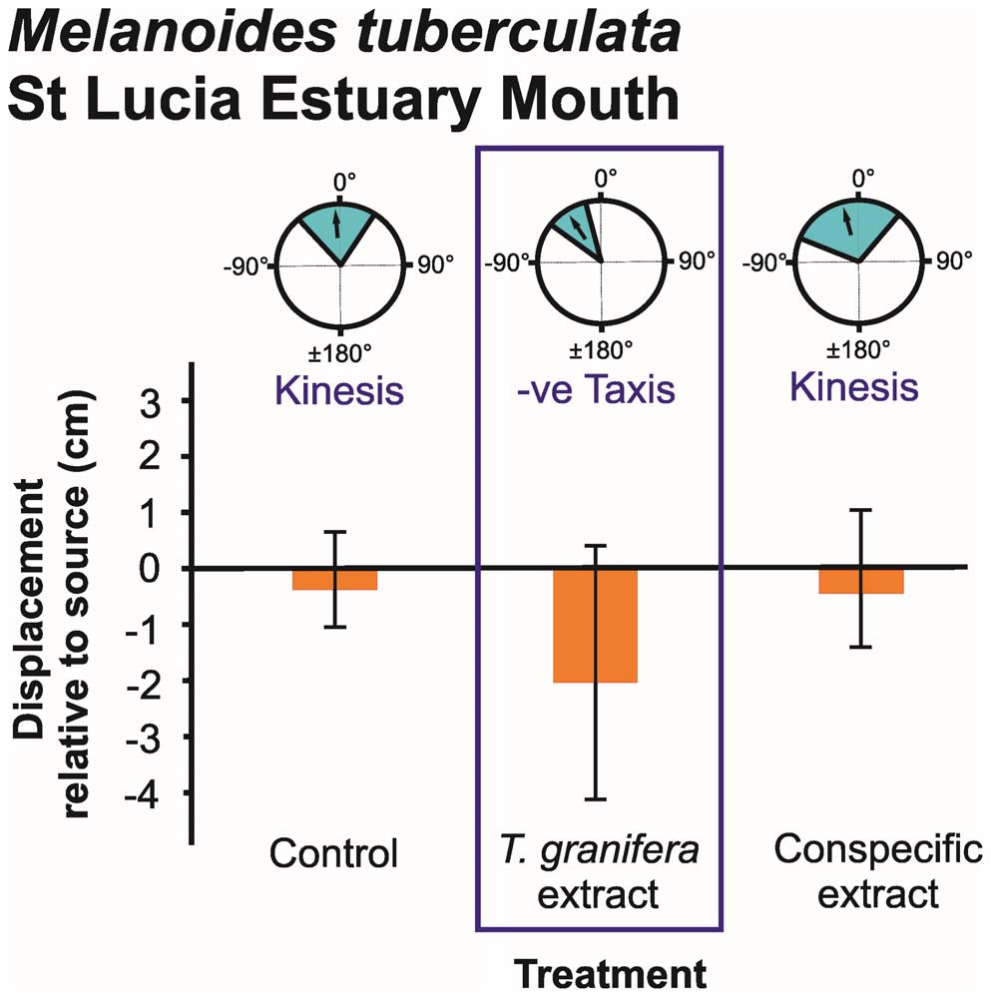
*Melanoides tuberculata* orientation and movement responses to different chemical cue treatments at St Lucia Estuary Mouth. Three types of data are presented: **refer to Fig. 2**. Treatments where a negative (−ve) taxis response occurred are demarcated. Individuals were tracked over a period of three minutes.

**Figure 6:**
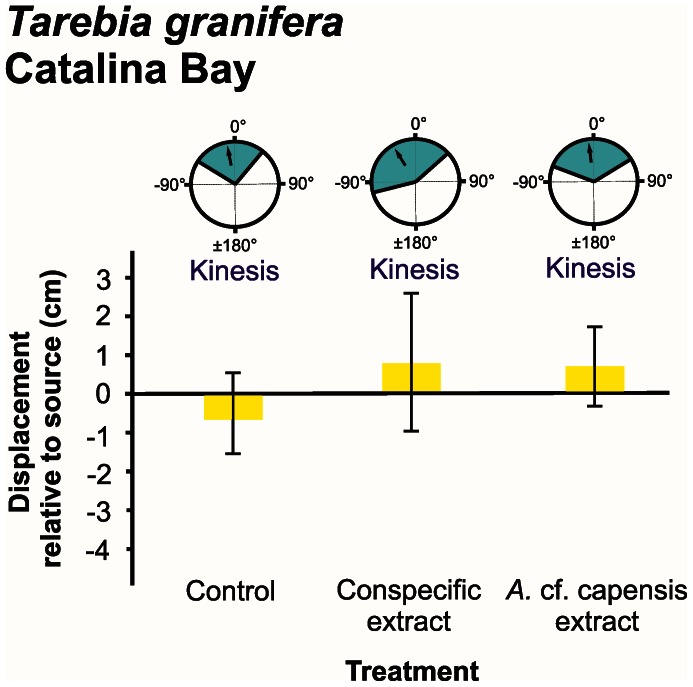
*Tarebia granifera* orientation and movement responses to different chemical cue treatments at Catalina Bay. Three types of data are presented: **refer to Fig. 2**. Individuals were tracked over a period of three minutes.

Responses were measured in terms of distribution of turning angles, displacement and number of steps taken (Figs. 2, 3, 4, 5, 6).

The mean turning angle of all native species was only significantly different from zero in response to the *Tarebia granifera* cue treatment: *Assiminea* cf. *capensis* at Catalina Bay (*t* = −4.894, d.f. = 8, *P* = 0.001) (Fig. 2); *A.* cf. *capensis* at Charter’s Creek (*t* = −4.221, d.f. = 9, *P = *0.002) (Fig. 3); *Coriandria durbanensis* at Lister’s Point (*t* = −5.335, d.f. = 5, *P* = 0.003) (Fig. 4); and *Melanoides tuberculata* at the St Lucia Mouth (*t* = −4.664, d.f. = 9, *P* = 0.001) (Fig. 5). The mean turning angle of the non-native *T. granifera* (Fig. 6) was not significantly different from zero for any of the treatments (*t–*tests, d.f. = 5, *P*>0.450). Mean turning angle was always negative for the *T. granifera* cue treatment (i.e. turned away from source, Figs. 2, 3, 4, 5), except when *T. granifera* was the test subject.

An analysis of variance of the mean displacement of gastropods produced a significant difference between treatments (*F*_2, 145_ = 5.533, *P* = 0.031) (Table S1 in File S1). Tukey’s HSD Post-hoc test revealed that the *T. granifera* extract treatment resulted in a significantly different (*P*<0.05) and negative (i.e. away from the source) movement response by all of the native gastropod species (Tables S2, S3, S4, S5, S6, S7, S8, S9, S10, S11, S12 in File S1), except *M. tuberculata* which displayed a negative but not significant (*F*_2, 29_ = 2.488, *P* = 0.102) movement response (Fig. 5, Table S7 in File S1). There were also no significant differences, in terms of displacement between all treatments for *T. granifera* (*F*_2, 29_ = 3.334, *P* = 0.051) (Table S5 in File S1).

An analysis of the mean number of steps taken by gastropods revealed no overall differences between treatments (*F*_2, 145_ = 2.010, *P* = 0.196) (Table S13 in File S1), except for *A.* cf. *capensis* at Charter’s Creek (*F*_2, 29_ = 5.763, *P = *0.008) where the mean number of steps was higher for the *T. granifera* extract treatment than for the others (Fig. 3, Tables S23 and S24 in File S1).

## Discussion

Historically, chemoreception studies have emphasized the identification of the chemical compound that stimulated a behavioural or physiological response. Consequently, a large proportion of substances have been identified as stimulants including those which promote or deter feeding, promote metamorphosis, evoke predator avoidance, attract mates or influence habitat selection [36], [37]. In contrast, the majority of studies that focus on interspecific interactions mediated by chemical cues have rarely identified the chemical structure of the cues themselves [20], [25], [38].

Although this study has not identified, isolated and purified the chemical cue in question through biochemical assays, the significant quantified behavioural responses suggest that the interspecific interaction among *Tarebia granifera* and native gastropods is driven by chemical cues. Metabolic wastes generally cause avoidance responses [36]; however preliminary results from the inorganic nutrient analysis revealed no significant difference in ammonium levels between extracts. It is therefore unlikely that the extracts contained cues derived from high levels of nitrogenous wastes, which would cause an avoidance response in all gastropod species. The results of these and further nutrient analyses are part of a separate study which is being prepared for publication. A more probable explanation is that the avoidance responses are caused by a secondary metabolite, possibly a biogenic volatile organic compound (VOC), released either by *T*. *granifera* itself or by microbial epibionts. VOCs are acknowledged as important infochemicals, most notably for their role in mediating interactions between plants and herbivores in terrestrial systems, however their ecological significance in the aquatic environment has become more recently recognized [39]. VOCs have been reported to mediate interspecific interactions [40], where they function as kairomones with only the recipient benefitting from the interaction [41]. However, there is a need for further research on other interactions which are potentially mediated by VOCs in the aquatic environment [39].

The behavioural responses, quantified as three components of movement: mean displacement, mean number of steps taken and mean turning angle, illustrate how chemical cues released by the non-native *T. granifera* affect native gastropod species (Figs. 2, 3, 4, 5). All gastropods displayed kinesis to orient in response to the control and respective conspecific treatments (Figs. 2, 3, 4, 5, 6). This indicates that movement in response to general environmental cues (control treatment), as well as chemical cues released by conspecifics, is random and undirected [35]. *T. granifera* responded in a similar way to the source of chemical cues released by the native *Assiminea* cf. *capensis* (Fig. 6). However, all three native species (Figs. 2, 3, 4, 5), displayed negative taxis to orient their movement in response to chemical cues released by the non-native *T. granifera*. This indicates that the movement of all three native species in response to chemical cues released by *T. granifera* was non-random [35] and in a direction away from the source. Remarkably, the same response was found in *A.* cf. *capensis* individuals from Charter’s Creek, *Coriandria durbanensis* individuals from Lister’s Point and *Melanoides tuberculata* from the St Lucia Mouth, none of which had any physical pre-exposure to *T. granifera* individuals.

*Coriandria durbanensis* individuals were generally more active than the other two native species (Fig. 4). This is unlikely to be due to the average temperature prevailing during the experiments, which was similar between sites for *C. durbanensis* at Lister’s Point (21.3°C) and *A.* cf. *capensis* at Catalina Bay (22.2°C). The highest temperature recorded was in March at Charter’s Creek (27.1°C) while the lowest was in July at the St Lucia Estuary Mouth (18.5°C). *A.* cf. *capensis* individuals from Catalina Bay (Fig. 2) took fewer steps on average and tended to move towards, and stay close to, the source, when exposed to the conspecific treatment, suggesting an aggregation response [16], [42]. In contrast, *A.* cf. *capensis* individuals at Charter’s Creek took more steps on average, and tended to move away and stay away from the source, in response to the *T. granifera* chemical cue treatment (Fig. 3).

Response to the control treatment in terms of displacement varied among species and was dependent on conditions at the site during the experiment. *T. granifera* individuals were observed to move closer to the source of chemical cues released by conspecifics and the native *A.* cf. *capensis,* which is currently still present at Catalina Bay (Fig. 6). Gastropods that co-habit areas have exhibited movement as a response to heterospecific alarm cues [43], which assists in predator avoidance. However, the response of *T. granifera* in this case could contribute towards a more directional range expansion towards areas already inhabited by native gastropods. These behavioural responses involving both orientation and movement away, in a non-random directed manner, from the source of a chemical cue released by the non-native *T. granifera,* may have direct consequences for the spatial distribution of native gastropods.

The release of chemical cues can cause heterospecifics to move away from an area. This may explain how *T. granifera* displaces native species. However, since native hetrospecifics were not present in any of the sites during the course of this study, their effect could not be tested. Therefore, explicit conclusions cannot be made about the mechanism at work in interactions between non-native and native species unless a native hetrospecific treatment is included in the analyses. Our understanding of the biotic interactions among different species is still incomplete, especially concerning aquatic organisms. Further studies are needed to identify the biochemical properties of the chemical cue released by *T. granifera,* and possibly also by other heterospecifics, which lead to avoidance responses in gastropod species.

## Supporting Information

File S1 **Table S1**. Results of ANOVA testing for differences in snail (n = 145) displacement between experimental treatments at St Lucia Estuary. **Table S2.** Results of Tukey HSD testing for differences in snail (n = 145) displacement among experimental treatments at St Lucia Estuary. **Table S3.** Results of ANOVA testing for differences in *Assiminea* cf. *capensis* (n = 29) displacement between experimental treatments at Catalina Bay. **Table S4.** Results of Tukey HSD testing for differences in *Assiminea* cf. *capensis* (n = 29) displacement among experimental treatments at Catalina Bay. **Table S5.** Results of ANOVA testing for differences in *Tarebia granifera* (n = 29) displacement between experimental treatments at Catalina Bay. **Table S6.** Results of Tukey HSD testing for differences in *Tarebia granifera* (n = 29) displacement among experimental treatments at Catalina Bay. **Table S7.** Results of ANOVA testing for differences in *Melanoides tuberculata* (n = 29) displacement between experimental treatments at St Lucia Estuary Mouth. **Table S8.** Results of Tukey HSD testing for differences in *Melanoides tuberculata* (n = 29) displacement among experimental treatments at St Lucia Estuary Mouth. **Table S9.** Results of ANOVA testing for differences in *Coriandria durbanensis* (n = 29) displacement between experimental treatments at Lister’s Point. **Table S10.** Results of Tukey HSD testing for differences in *Coriandria durbanensis* (n = 29) displacement among experimental treatments at Lister’s Point. **Table S11.** Results of ANOVA testing for differences in *Assiminea* cf. *capensis* (n = 29) displacement between experimental treatments at Charter’s Creek. **Table S12.** Results of Tukey HSD testing for differences in *Assiminea* cf. *capensis* (n = 29) displacement among experimental treatments at Charter’s Creek. **Table S13.** Results of ANOVA testing for differences in average number of steps taken by snails (n = 145), between experimental treatments at St Lucia Estuary. **Table S14.** Results of Tukey HSD testing for differences in average number of steps taken by snails (n = 145), among experimental treatments at St Lucia Estuary. **Table S15.** Results of ANOVA testing for differences in average number of steps taken by *Assiminea* cf. *capensis* (n = 29), between experimental treatments at Catalina Bay. **Table S16.** Results of Tukey HSD testing for differences in average number of steps taken by *Assiminea* cf. *capensis* (n = 29), among experimental treatments at Catalina Bay. **Table S17.** Results of ANOVA testing for differences in average number of steps taken by *Tarebia granifera* (n = 29), between experimental treatments at Catalina Bay. **Table S18.** Results of Tukey HSD testing for differences in average number of steps taken by *Tarebia granifera* (n = 29), among experimental treatments at Catalina Bay. **Table S19.** Results of ANOVA testing for differences in average number of steps taken by *Melanoides tuberculata* (n = 29), between experimental treatments at St Lucia Estuary Mouth. **Table S20.** Results of Tukey HSD testing for differences in average number of steps taken by *Melanoides tuberculata* (n = 29), among experimental treatments at St Lucia Estuary Mouth. **Table S21.** Results of ANOVA testing for differences in average number of steps taken by *Coriandria durbanensis* (n = 29), between experimental treatments at Lister’s Point. **Table S22.** Results of Tukey HSD testing for differences in average number of steps taken by *Coriandria durbanensis* (n = 29), among experimental treatments at Lister’s Point. **Table S23.** Results of ANOVA testing for differences in average number of steps taken by *Assiminea* cf. *capensis* (n = 29), between experimental treatments at Charter’s Creek. **Table S24.** Results of Tukey HSD testing for differences in average number of steps taken by *Assiminea* cf. *capensis* (n = 29), among experimental treatments at Charter’s Creek.
(DOC)
