# Supplementary material for: Chemical Cues Released by an Alien Invasive Aquatic Gastropod Drive Its Invasion Success
Source: PLoS One. 2013 May 15;8(5):e64071. doi: 10.1371/journal.pone.0064071 (PMC3654916; doi:10.1371/journal.pone.0064071)
Supplement: File S1 — Table S1. Results of ANOVA testing for differences in snail (n = 145) displacement between experimental treatments at St Lucia Estuary. Table S2. Results of Tukey HSD testing for differences in snail (n = 145) displacement among experimental treatments at St Lucia Estuary. Table S3. Results of ANOVA testing for differences in Assiminea cf. capensis (n = 29) displacement between experimental treatments at Catalina Bay. Table S4. Results of Tukey HSD testing for differences in Assiminea cf. capensis (n = 29) displacement among experimental treatments at Catalina Bay. Table S5. Results of ANOVA testing for differences in Tarebia granifera (n = 29) displacement between experimental treatments at Catalina Bay. Table S6. Results of Tukey HSD testing for differences in Tarebia granifera (n = 29) displacement among experimental treatments at Catalina Bay. Table S7. Results of ANOVA testing for differences in Melanoides tuberculata (n = 29) displacement between experimental treatments at St Lucia Estuary Mouth. Table S8. Results of Tukey HSD testing for differences in Melanoides tuberculata (n = 29) displacement among experimental treatments at St Lucia Estuary Mouth. Table S9. Results of ANOVA testing for differences in Coriandria durbanensis (n = 29) displacement between experimental treatments at Lister’s Point. Table S10. Results of Tukey HSD testing for differences in Coriandria durbanensis (n = 29) displacement among experimental treatments at Lister’s Point. Table S11. Results of ANOVA testing for differences in Assiminea cf. capensis (n = 29) displacement between experimental treatments at Charter’s Creek. Table S12. Results of Tukey HSD testing for differences in Assiminea cf. capensis (n = 29) displacement among experimental treatments at Charter’s Creek. Table S13. Results of ANOVA testing for differences in average number of steps taken by snails (n = 145), between experimental treatments at St Lucia Estuary. Table S14. Results of Tukey HSD testing for difference [file pone.0064071.s001.doc]

| **Table S1. Results of ANOVA testing for differences in snail (n = 145) displacement between experimental treatments at St Lucia Estuary.**   | **Source** |  | **Type III Sum of Squares** | **df** | **Mean Square** | **F** | **Sig.** | | --- | --- | --- | --- | --- | --- | --- | | Treatment | Hypothesis | 84.288 | 2 | 42.144 | 5.533 | .031 | |  | Error | 355.133 | 142 | 2.732 |  |  |   **Table S2. Results of Tukey HSD testing for differences in snail (n = 145) displacement among experimental treatments at St Lucia Estuary.** | | | | | | |
| --- | --- | --- | --- | --- | --- | --- |
| **(I) Treatment** | **(J) Treatment** | **Mean Difference (I-J)** | **Std. Error** | **Sig.** | **95% Confidence Interval** | |
|  |  |  |  |  | **Lower Bound** | **Upper Bound** |
| Conspec | Control | .2729 | .33399 | .693 | -.5190 | 1.0647 |
|  | Tarebia | 1.7106^*^ | .33580 | .000 | .9144 | 2.5067 |
| Control | Conspec | -.2729 | .33399 | .693 | -1.0647 | .5190 |
|  | Tarebia | 1.4377^*^ | .33917 | .000 | .6336 | 2.2418 |
| Tarebia | Conspec | -1.7106^*^ | .33580 | .000 | -2.5067 | -.9144 |
|  | Control | -1.4377^*^ | .33917 | .000 | -2.2418 | -.6336 |

Based on observed means.

The error term is Mean Square(Error) = 2.732.

* The mean difference is significant at the 0.05 level.

**Table S3. Results of ANOVA testing for differences in *Assiminea* cf. *capensis* (n = 29) displacement between experimental treatments at Catalina Bay.**

| **Source** |  | **Type III Sum of Squares** | **df** | **Mean Square** | **F** | **Sig.** |
| --- | --- | --- | --- | --- | --- | --- |
| Treatment | Hypothesis | 31.666 | 2 | 15.833 | 6.551 | .005 |
|  | Error | 62.840 | 26 | 2.417 |  |  |

**Table S4. Results of Tukey HSD testing for differences in *Assiminea* cf. *capensis* (n = 29) displacement among experimental treatments at Catalina Bay.**

| **(I) Treatment** | **(J) Treatment** | **Mean Difference (I-J)** | **Std. Error** | **Sig.** | **95% Confidence Interval** | |
| --- | --- | --- | --- | --- | --- | --- |
|  |  |  |  |  | **Lower Bound** | **Upper Bound** |
| Conspec | Control | .2333 | .71431 | .943 | -1.5417 | 2.0083 |
|  | Tarebia | 2.3000^*^ | .69526 | .007 | .5724 | 4.0276 |
| Control | Conspec | -.2333 | .71431 | .943 | -2.0083 | 1.5417 |
|  | Tarebia | 2.0667^*^ | .71431 | .020 | .2917 | 3.8417 |
| Tarebia | Conspec | -2.3000^*^ | .69526 | .007 | -4.0276 | -.5724 |
|  | Control | -2.0667^*^ | .71431 | .020 | -3.8417 | -.2917 |

Based on observed means.

The error term is Mean Square(Error) = 2.417.

* The mean difference is significant at the 0.05 level.

**Table S5. Results of ANOVA testing for differences in *Tarebia granifera* (n = 29) displacement between experimental treatments at Catalina Bay.**

| **Source** |  | **Type III Sum of Squares** | **df** | **Mean Square** | **F** | **Sig.** |
| --- | --- | --- | --- | --- | --- | --- |
| Treatment | Hypothesis | 10.690 | 2 | 5.345 | 3.334 | .051 |
|  | Error | 41.680 | 26 | 1.603 |  |  |

**Table S6. Results of Tukey HSD testing for differences in *Tarebia granifera* (n = 29) displacement among experimental treatments at Catalina Bay.**

| **(I) Treatment** | **(J) Treatment** | **Mean Difference (I-J)** | **Std. Error** | **Sig.** | **95% Confidence Interval** | |
| --- | --- | --- | --- | --- | --- | --- |
|  |  |  |  |  | **Lower Bound** | **Upper Bound** |
| Conspec | Control | 1.2000 | .58175 | .118 | -.2456 | 2.6456 |
|  | Tarebia | -.2000 | .56623 | .934 | -1.6070 | 1.2070 |
| Control | Conspec | -1.2000 | .58175 | .118 | -2.6456 | .2456 |
|  | Tarebia | -1.4000 | .58175 | .059 | -2.8456 | .0456 |
| Tarebia | Conspec | .2000 | .56623 | .934 | -1.2070 | 1.6070 |
|  | Control | 1.4000 | .58175 | .059 | -.0456 | 2.8456 |

Based on observed means.

The error term is Mean Square(Error) = 1.603.

**Table S7. Results of ANOVA testing for differences in *Melanoides tuberculata* (n = 29) displacement between experimental treatments at St Lucia Estuary Mouth.**

| **Source** |  | **Type III Sum of Squares** | **df** | **Mean Square** | **F** | **Sig.** |
| --- | --- | --- | --- | --- | --- | --- |
| Treatment | Hypothesis | 10.931 | 2 | 5.465 | 2.488 | .102 |
|  | Error | 59.316 | 26 | 2.197 |  |  |

**Table S8. Results of Tukey HSD testing for differences in *Melanoides tuberculata* (n = 29) displacement among experimental treatments at St Lucia Estuary Mouth.**

| **(I) Treatment** | **(J) Treatment** | **Mean Difference (I-J)** | **Std. Error** | **Sig.** | **95% Confidence Interval** | |
| --- | --- | --- | --- | --- | --- | --- |
|  |  |  |  |  | **Lower Bound** | **Upper Bound** |
| Conspec | Control | 1.2000 | .58175 | .118 | -.2456 | 2.6456 |
|  | Tarebia | -.2000 | .56623 | .934 | -1.6070 | 1.2070 |
| Control | Conspec | -1.2000 | .58175 | .118 | -2.6456 | .2456 |
|  | Tarebia | -1.4000 | .58175 | .059 | -2.8456 | .0456 |
| Tarebia | Conspec | .2000 | .56623 | .934 | -1.2070 | 1.6070 |
|  | Control | 1.4000 | .58175 | .059 | -.0456 | 2.8456 |

Based on observed means.

The error term is Mean Square(Error) = 1.603.

**Table S9. Results of ANOVA testing for differences in *Coriandria durbanensis* (n = 29) displacement between experimental treatments at Lister’s Point**.

| **Source** |  | **Type III Sum of Squares** | **df** | **Mean Square** | **F** | **Sig.** |
| --- | --- | --- | --- | --- | --- | --- |
| Treatment | Hypothesis | 36.335 | 2 | 18.168 | 5.383 | .012 |
|  | Error | 81.003 | 26 | 3.375 |  |  |

**Table S10. Results of Tukey HSD testing for differences in *Coriandria durbanensis* (n = 29) displacement among experimental treatments at Lister’s Point.**

| **(I) Treatment** | **(J) Treatment** | **Mean Difference (I-J)** | **Std. Error** | **Sig.** | **95% Confidence Interval** | |
| --- | --- | --- | --- | --- | --- | --- |
|  |  |  |  |  | **Lower Bound** | **Upper Bound** |
| Conspec | Control | -.2800 | .82160 | .938 | -2.3318 | 1.7718 |
|  | Tarebia | 2.4929^*^ | .90536 | .029 | .2319 | 4.7538 |
| Control | Conspec | .2800 | .82160 | .938 | -1.7718 | 2.3318 |
|  | Tarebia | 2.7729^*^ | .90536 | .014 | .5119 | 5.0338 |
| Tarebia | Conspec | -2.4929^*^ | .90536 | .029 | -4.7538 | -.2319 |
|  | Control | -2.7729^*^ | .90536 | .014 | -5.0338 | -.5119 |

Based on observed means.

The error term is Mean Square(Error) = 3.375.

* The mean difference is significant at the 0.05 level.

**Table S11. Results of ANOVA testing for differences in *Assiminea* cf. *capensis* (n = 29) displacement between experimental treatments at Charter’s Creek**.

| **Source** |  | **Type III Sum of Squares** | **df** | **Mean Square** | **F** | **Sig.** |
| --- | --- | --- | --- | --- | --- | --- |
| Treatment | Hypothesis | 52.631 | 2 | 26.316 | 6.442 | .005 |
|  | Error | 110.294 | 26 | 4.085 |  |  |

**Table S12. Results of Tukey HSD testing for differences in *Assiminea* cf. *capensis* (n = 29) displacement among experimental treatments at Charter’s Creek.**

| **(I) Treatment** | **(J) Treatment** | **Mean Difference (I-J)** | **Std. Error** | **Sig.** | **95% Confidence Interval** | |
| --- | --- | --- | --- | --- | --- | --- |
|  |  |  |  |  | **Lower Bound** | **Upper Bound** |
| Conspec | Control | .2700 | .90387 | .952 | -1.9711 | 2.5111 |
|  | Tarebia | 2.9350^*^ | .90387 | .008 | .6939 | 5.1761 |
| Control | Conspec | -.2700 | .90387 | .952 | -2.5111 | 1.9711 |
|  | Tarebia | 2.6650^*^ | .90387 | .017 | .4239 | 4.9061 |
| Tarebia | Conspec | -2.9350^*^ | .90387 | .008 | -5.1761 | -.6939 |
|  | Control | -2.6650^*^ | .90387 | .017 | -4.9061 | -.4239 |

Based on observed means.

The error term is Mean Square(Error) = 4.085.

* The mean difference is significant at the 0.05 level.

**Table S13. Results of ANOVA testing for differences in average number of steps taken by snails (n = 145), between experimental treatments at St Lucia Estuary.**

| **Source** |  | **Type III Sum of Squares** | **df** | **Mean Square** | **F** | **Sig.** |
| --- | --- | --- | --- | --- | --- | --- |
| Treatment | Hypothesis | 57.564 | 2 | 28.782 | 2.010 | .196 |
|  | Error | 1935.084 | 142 | 14.885 |  |  |

**Table S14. Results of Tukey HSD testing for differences in average number of steps taken by snails (n = 145), among experimental treatments at St Lucia Estuary.**

| **(I) Treatment** | **(J) Treatment** | **Mean Difference (I-J)** | **Std. Error** | **Sig.** | **95% Confidence Interval** | |
| --- | --- | --- | --- | --- | --- | --- |
|  |  |  |  |  | **Lower Bound** | **Upper Bound** |
| Conspec | Control | -.2583 | .77962 | .941 | -2.1067 | 1.5900 |
|  | Tarebia | -1.3426 | .78384 | .204 | -3.2009 | .5158 |
| Control | Conspec | .2583 | .77962 | .941 | -1.5900 | 2.1067 |
|  | Tarebia | -1.0842 | .79172 | .360 | -2.9613 | .7928 |
| Tarebia | Conspec | 1.3426 | .78384 | .204 | -.5158 | 3.2009 |
|  | Control | 1.0842 | .79172 | .360 | -.7928 | 2.9613 |

Based on observed means.

The error term is Mean Square(Error) = 14.885.

**Table S15. Results of ANOVA testing for differences in average number of steps taken by *Assiminea* cf. *capensis* (n = 29), between experimental treatments at Catalina Bay.**

| **Source** |  | **Type III Sum of Squares** | **df** | **Mean Square** | **F** | **Sig.** |
| --- | --- | --- | --- | --- | --- | --- |
| Treatment | Hypothesis | 39.957 | 2 | 19.979 | 2.856 | .076 |
|  | Error | 174.900 | 26 | 6.996 |  |  |

**Table S16. Results of Tukey HSD testing for differences in average number of steps taken by *Assiminea* cf. *capensis* (n = 29),** among experimental treatments at Catalina Bay.

| **(I) Treatment** | **(J) Treatment** | **Mean Difference (I-J)** | **Std. Error** | **Sig.** | **95% Confidence Interval** | |
| --- | --- | --- | --- | --- | --- | --- |
|  |  |  |  |  | **Lower Bound** | **Upper Bound** |
| Conspec | Control | -1.2333 | 1.21529 | .575 | -4.2604 | 1.7938 |
|  | Tarebia | -2.9000 | 1.21529 | .062 | -5.9271 | .1271 |
| Control | Conspec | 1.2333 | 1.21529 | .575 | -1.7938 | 4.2604 |
|  | Tarebia | -1.6667 | 1.24686 | .389 | -4.7724 | 1.4391 |
| Tarebia | Conspec | 2.9000 | 1.21529 | .062 | -.1271 | 5.9271 |
|  | Control | 1.6667 | 1.24686 | .389 | -1.4391 | 4.7724 |

Based on observed means.

The error term is Mean Square(Error) = 6.996.

**Table S17. Results of ANOVA testing for differences in average number of steps taken by *Tarebia granifera* (n = 29), between experimental treatments at Catalina Bay.**

| **Source** |  | **Type III Sum of Squares** | **df** | **Mean Square** | **F** | **Sig.** |
| --- | --- | --- | --- | --- | --- | --- |
| Treatment | Hypothesis | 6.582 | 2 | 3.291 | .397 | .676 |
|  | Error | 215.556 | 26 | 8.291 |  |  |

**Table S18. Results of Tukey HSD testing for differences in average number of steps taken by *Tarebia granifera* (n = 29), among experimental treatments at Catalina Bay.**

| **(I) Treatment** | **(J) Treatment** | **Mean Difference (I-J)** | **Std. Error** | **Sig.** | **95% Confidence Interval** | |
| --- | --- | --- | --- | --- | --- | --- |
|  |  |  |  |  | **Lower Bound** | **Upper Bound** |
| Conspec | Control | -.5778 | 1.32297 | .901 | -3.8652 | 2.7097 |
|  | Tarebia | .6000 | 1.28768 | .888 | -2.5997 | 3.7997 |
| Control | Conspec | .5778 | 1.32297 | .901 | -2.7097 | 3.8652 |
|  | Tarebia | 1.1778 | 1.32297 | .651 | -2.1097 | 4.4652 |
| Tarebia | Conspec | -.6000 | 1.28768 | .888 | -3.7997 | 2.5997 |
|  | Control | -1.1778 | 1.32297 | .651 | -4.4652 | 2.1097 |

Based on observed means.

The error term is Mean Square(Error) = 8.291.

**Table S19. Results of ANOVA testing for differences in average number of steps taken by *Melanoides tuberculata* (n = 29), between experimental treatments at St Lucia Estuary Mouth**.

| **Source** |  | **Type III Sum of Squares** | **df** | **Mean Square** | **F** | **Sig.** |
| --- | --- | --- | --- | --- | --- | --- |
| Treatment | Hypothesis | 33.800 | 2 | 16.900 | 1.317 | .285 |
|  | Error | 346.500 | 26 | 12.833 |  |  |

**Table S20. Results of Tukey HSD testing for differences in average number of steps taken by *Melanoides tuberculata* (n = 29), among experimental treatments at St Lucia Estuary Mouth.**

| **(I) Treatment** | **(J) Treatment** | **Mean Difference (I-J)** | **Std. Error** | **Sig.** | **95% Confidence Interval** | |
| --- | --- | --- | --- | --- | --- | --- |
|  |  |  |  |  | **Lower Bound** | **Upper Bound** |
| Conspec | Control | -.1000 | 1.60208 | .998 | -4.0722 | 3.8722 |
|  | Tarebia | -2.3000 | 1.60208 | .337 | -6.2722 | 1.6722 |
| Control | Conspec | .1000 | 1.60208 | .998 | -3.8722 | 4.0722 |
|  | Tarebia | -2.2000 | 1.60208 | .369 | -6.1722 | 1.7722 |
| Tarebia | Conspec | 2.3000 | 1.60208 | .337 | -1.6722 | 6.2722 |
|  | Control | 2.2000 | 1.60208 | .369 | -1.7722 | 6.1722 |

Based on observed means.

The error term is Mean Square(Error) = 12.833.

**Table S21. Results of ANOVA testing for differences in average number of steps taken by *Coriandria durbanensis* (n = 29), between experimental treatments at Lister’s Point.**

| **Source** |  | **Type III Sum of Squares** | **df** | **Mean Square** | **F** | **Sig.** |
| --- | --- | --- | --- | --- | --- | --- |
| Treatment | Hypothesis | 9.800 | 2 | 4.900 | .142 | .868 |
|  | Error | 932.900 | 26 | 34.552 |  |  |

**Table S22. Results of Tukey HSD testing for differences in average number of steps taken by *Coriandria durbanensis* (n = 29), among experimental treatments at Lister’s Point.**

| **(I) Treatment** | **(J) Treatment** | **Mean Difference (I-J)** | **Std. Error** | **Sig.** | **95% Confidence Interval** | |
| --- | --- | --- | --- | --- | --- | --- |
|  |  |  |  |  | **Lower Bound** | **Upper Bound** |
| Conspec | Control | 1.4000 | 2.62876 | .856 | -5.1178 | 7.9178 |
|  | Tarebia | .7000 | 2.62876 | .962 | -5.8178 | 7.2178 |
| Control | Conspec | -1.4000 | 2.62876 | .856 | -7.9178 | 5.1178 |
|  | Tarebia | -.7000 | 2.62876 | .962 | -7.2178 | 5.8178 |
| Tarebia | Conspec | -.7000 | 2.62876 | .962 | -7.2178 | 5.8178 |
|  | Control | .7000 | 2.62876 | .962 | -5.8178 | 7.2178 |

Based on observed means.

The error term is Mean Square(Error) = 34.552.

**Table S23. Results of ANOVA testing for differences in average number of steps taken by *Assiminea* cf. *capensis* (n = 29), between experimental treatments at Charter’s Creek**.

| **Source** |  | **Type III Sum of Squares** | **df** | **Mean Square** | **F** | **Sig.** |
| --- | --- | --- | --- | --- | --- | --- |
| Treatment | Hypothesis | 105.267 | 2 | 52.633 | 5.763 | .008 |
|  | Error | 246.600 | 27 | 9.133 |  |  |

**Table S24. Results of Tukey HSD testing for differences in average number of steps taken by *Assiminea* cf. *capensis* (n = 29), among experimental treatments at Charter’s Creek.**

| **(I) Treatment** | **(J) Treatment** | **Mean Difference (I-J)** | **Std. Error** | **Sig.** | **95% Confidence Interval** | |
| --- | --- | --- | --- | --- | --- | --- |
|  |  |  |  |  | **Lower Bound** | **Upper Bound** |
| Conspec | Control | -.5000 | 1.35154 | .928 | -3.8510 | 2.8510 |
|  | Tarebia | -4.2000^*^ | 1.35154 | .012 | -7.5510 | -.8490 |
| Control | Conspec | .5000 | 1.35154 | .928 | -2.8510 | 3.8510 |
|  | Tarebia | -3.7000^*^ | 1.35154 | .028 | -7.0510 | -.3490 |
| Tarebia | Conspec | 4.2000^*^ | 1.35154 | .012 | .8490 | 7.5510 |
|  | Control | 3.7000^*^ | 1.35154 | .028 | .3490 | 7.0510 |

Based on observed means.

The error term is Mean Square(Error) = 9.133.

* The mean difference is significant at the 0.05 level.
